# Genome-wide screening of lipoproteins in *Actinobacillus pleuropneumoniae* identifies three antigens that confer protection against virulent challenge

**DOI:** 10.1038/s41598-020-58968-7

**Published:** 2020-02-11

**Authors:** Yurou Cao, Lulu Gao, Li Zhang, Lixiang Zhou, Jihong Yang, Lingfu Deng, Jin Zhao, Chao Qi, Jinlin Liu

**Affiliations:** Hubei Key Laboratory of Genetic Regulation and Integrative Biology, College of Life Sciences, Central China Normal University, Wuhan, Hubei 430079 China

**Keywords:** Protein vaccines, Protein vaccines

## Abstract

*Actinobacillus pleuropneumoniae* is an important veterinary pathogen that causes porcine pleuropneumonia. Lipoproteins of bacterial pathogens play pleiotropic roles in the infection process. In addition, many bacterial lipoproteins are antigenic and immunoprotective. Therefore, characterization of lipoproteins is a promising strategy for identification of novel vaccine candidates or diagnostic markers. We cloned 58 lipoproteins from *A*. *pleuropneumoniae* JL03 (serovar 3) and expressed them in *Escherichia coli*. Five proteins with strong positive signals in western blotting analysis were used to immunize mice. These proteins elicited significant antibody responses, and three of them (APJL_0922, APJL_1380 and APJL_1976) generated efficient immunoprotection in mice against lethal heterologous challenge with *A*. *pleuropneumoniae* 4074 (serovar 1), both in the active and passive immunization assays. Then immunogenicity of these three lipoproteins (APJL_0922, APJL_1380 and APJL_1976) were further tested in pigs. Results showed that these proteins elicited considerable humoral immune responses and effective protective immunity against virulent *A*. *pleuropneumoniae* challenge. Our findings suggest that these three novel lipoproteins could be potential subunit vaccine candidates.

## Introduction

*Actinobacillus pleuropneumoniae* is a Gram-negative bacterium that colonizes the upper respiratory tract of pigs, and causes porcine pleuropneumonia, a common respiratory infection that is characterized as acute hemorrhagic to chronic necrotic–fibrinous pleuropneumonia and associated with significant economic losses in the pig industry worldwide^1^. Great efforts have been devoted to the characterization and control of porcine pleuropneumonia in the past 30 years, and considerable achievements have been made. A total of 18 serovars of *A. pleuropneumoniae* have been identified till now, all of these serovars are able to colonize pigs^2^. Since *A. pleuropneumoniae* is susceptible to drying and other antibacterial factors, direct transmission from infected to susceptible healthy pigs is considered to be the most frequent means by which the disease spreads^3^. Airborne transmission of *A. pleuropneumoniae* was also confirmed to occur over a short distance (1 m)^4^. Pigs of all ages are susceptible to *A. pleuropneumoniae* infection, especially fattening pigs at about 3-months old, and the first outbreak in herds often leads to high morbidity and mortality^5^. *A. pleuropneumoniae* contains several virulence factors, which are involved in the steps of adherence, colonization and tissue damage during infection, including Apx toxins, LPS, CPS, adhesins, outer membrane proteins, and transcriptional regulators^6^.

Treatment of bacterial diseases of food-producing animals by extensive use of antimicrobial agents leads to antimicrobial resistance (AMR), a serious consequence that has attracted growing concerns worldwide^7^. The problem of AMR in *A. pleuropneumoniae* should not be underestimated at present^8^, and the use of drugs is undesirable for consumers of ethically produced food. Therefore, vaccination is a one of the most promising strategies for the control of porcine pleuropneumonia^9^. Several types of vaccines against this disease, including inactivated vaccine, subunit vaccine, ghost vaccine, DNA vaccine, edible vaccine, heterologous vaccine and live attenuated vaccine, have been developed in recent years^10^. However, only the first two types of vaccines have been introduced to the market, and other vaccines, which have been claimed to be effective, still have a long way to go before they can be marketed. Advantages of inactivated and subunit vaccines in the control of porcine pleuropneumonia are recognized, but some defects are notable. Bacterin-based inactivated vaccines often show limited cross-serovar protection, and subunit vaccines protect animals from death, but do not eradicate the invading bacteria completely^9^. Improvement in the immunogenicity of existing vaccines is a highly desired objective in this field. Bacterin formula, composed of more than one prevalent *A. pleuropneumoniae* serovar, could provide improved cross-serovar protection^11^. Discovery of novel conserved and protective antigens based on our understanding of *A. pleuropneumoniae* infection, or through modern proteomics technologies, is essential for the development of effective vaccines against porcine pleuropneumonia^12–14^.

Bacterial lipoproteins, typically characterized by lipid modification of cysteine at the end of the *N*-terminal lipo-box, are a set of membrane proteins with many different functions, including cellular structural maintenance, cell division, nutrient transport, signal transduction, and pathogenesis^15^. In addition, many bacterial lipoproteins are protective antigens and good candidates for vaccine development^16^. A number of lipoproteins were predicted from *A. pleuropneumoniae* in previous studies^17,18^. To evaluate the potential of these lipoproteins as vaccine candidates, 55 lipoproteins were cloned from the genome of *A. pleuropneumoniae* JL03 (serovar 3) and expressed in *Escherichia coli*. The reactivity of these recombinant proteins was assessed by western blotting analysis, and five of them were selected and tested for their potential as vaccine candidates against *A. pleuropneumoniae* infection in mice. Then immunogenicity and protective efficacy of three lipoproteins, APJL_0922, APJL_1380 and APJL_1976 were further tested in pigs.

## Results

### Cloning and expression of lipoproteins

**Figure 1:**
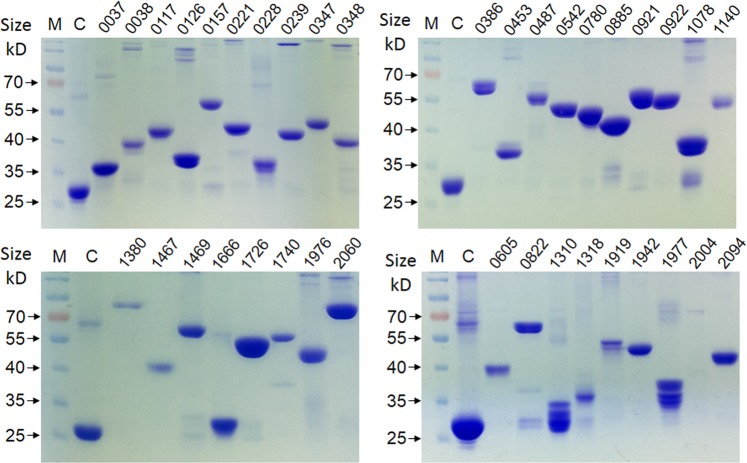
Purification of recombinant lipoproteins expressed in the supernatant of *E. coli* lysate. Recombinant proteins were purified from *E. coli* lysate using a glutathione–Sepharose 4B affinity chromatography column, separated by 12% SDS-PAGE and stained with Coomassie Brilliant Blue. Lane M, prestained protein ladder (Fermentas, Vilnius, Lithuania); lane C, recombinant GST control; other lanes: recombinant lipoproteins, the lane number indicates the protein number of *A. pleuropneumoniae* JL03.

Based on our previous bioinformatics prediction, 60 lipoproteins were identified from the *A. pleuropneumoniae* JL03 (Table 1) genome for further investigation. Since lipoproteins Lip40 and PalA have been analyzed before^18,19^, here, the remaining 58 open reading frames were amplified by PCRs (Table S1) and *E. coli* expression vectors were constructed, to generate recombinant proteins. Of the 58 genes cloned, 47 (81%) were expressed successfully. We found that 37 (79%) and 10 (21%) of these recombinant lipoproteins were soluble and insoluble when produced in *E. coli* (Table S2), respectively. The recombinant proteins in the supernatant were purified by affinity chromatography (Fig. 1).

**Table 1:** Bacterial strains and plasmids used in this study.

Strains, plasmids and primers	Relevant characteristics	Sources
*A. pleuropneumoniae*		
JL03	serovar 3	Field isolate
4074	serovar 1	From Dr. Pat Blackall
*E. coli*		
DH5a	Cloning vehicle: *supE44* △*lacU169* (*φ80 lacZ*△M15) *hsdR17 recA1 endA1 gyrA96 thi-1 relA1*	Takara
BL21(DE3)	Expression host: F^−^ *ompT* r^−^_B_ m^−^_B_; DE3 is a λ derivative carrying *lacI* and T7 RNA polymerase genes under placUV5 control	Takara
Plasmids		
pMD18-T	*E. coli* cloning vector carrying an ampicillin resistance determinant.	Takara
pMD-APJL_	pMD18-T carrying the coding sequence for lipoprotein of *A. pleuropneumoniae* JL03, for sequence analysis.	This work
pGEX-KG	N-terminal glutathione *S*-transferase (GST) fusion expression vector: pBR322 *ori*, Amp ^r^.	Ref. ^44^
pGEX-APJL_	pGEX-KG carrying the coding sequence for lipoprotein of *A. pleuropneumoniae* JL03, for the expression of GST-fusion recombinant protein	This work

### Identification of candidate proteins by western blotting

**Figure 2:**
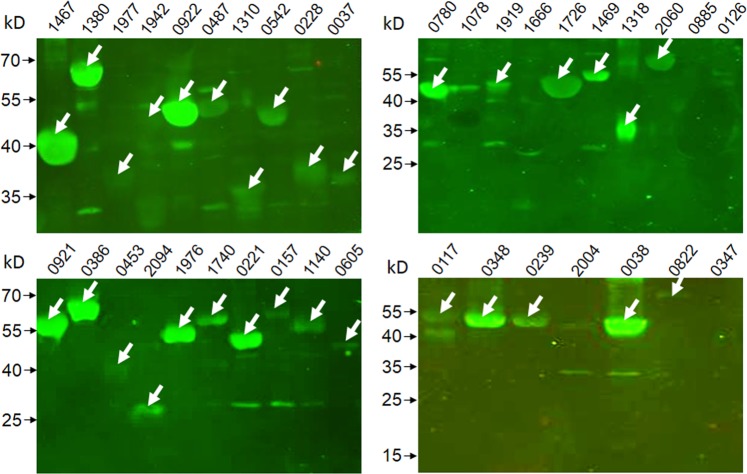
Immunoreactivity of soluble lipoproteins. Protein samples were separated by 12% SDS-PAGE and blotted onto nitrocellulose membranes. To test the serovar-cross reactivity of these lipoproteins cloned from *A. pleuropneumoniae* serovar 3, the membranes were incubated with rabbit polyclonal antibodies against *A. pleuropneumoniae* serovar 7, and Dylight-800-conjugated goat anti-rabbit IgG, sequentially, and images were viewed using a scanned infrared imaging system (Odyssey; LICOR). A white arrow indicates a specific signal for lipoprotein. The lane number indicates the protein number of *A. pleuropneumoniae* JL03.

To filter potential crossprotective recombinant proteins, we first verified whether these recombinant proteins could be recognized and combined by a heterologous antiserum against *A. pleuropneumoniae* serovar 7. Referring to the results of western blotting (Fig. 2), 31 of 37 tested soluble proteins yielded positive results. Information about these immunoreactive antigens is shown in Table 2. The proteins in Table 2 were categorized into three groups: (i) group 1 consisted of five proteins that had been studied previously for their roles in the *A. pleuropneumoniae* infection or in immunoprotection; (ii) group 2 consisted of seven proteins that had been mentioned in previous studies, or which belonged to well-described protein families in other species, but whose immunogenicity and function in *A. pleuropneumoniae* were unknown; and (iii) group 3 consisted of 19 proteins so far only annotated as conserved lipoproteins or hypothetical proteins. Five proteins, APJL_0386, APJL_0922, APJL_1380, APJL_1740 and APJL_1976, which showed strong positive reactions in western blotting, were selected for further evaluation as vaccine candidates.

**Table 2:** Information of the immunoreactive lipoproteins of *A. pleuropneumoniae.* ^*a*^ND, no homolog was found in the target genome.

Group	Lipoprotein in JL03				MW of recombinant protein	Homologs in L20 and AP76	
	locus_tag	protein_id	gene name	annotation		L20 locus_tag	AP76 locus_tag
I	APJL_0386	YP_001651421.1	*potD*	spermidine/putrescine ABC transporter periplasmic substrate-binding protein	64.0	APL_0368	APP7_0390
	APJL_0542	YP_001651561.1	*tadD*	Flp pilus assembly protein TadD	52.3	APL_0549	APP7_0590
	APJL_0780	YP_001651787.1	*lolB*	Outer membrane lipoprotein LolB	48.5	APL_0777	APP7_0838
	APJL_0921	YP_001651923.1	*plpB*	outer membrane lipoprotein 2	54.1	APL_0909	APP7_0969
	APJL_2060	YP_001653049.1	*hbpA2*	heme-binding protein A	84.7	APL_2010	APP7_2097
II	APJL_0038	YP_001651088.1	*slyB*	outer membrane lipoprotein	40.0	APL_0037	APP7_0037
	APJL_0157	YP_001651205.1	*apbE*	thiamine biosynthesis lipoprotein	62.0	APL_0156	APP7_0158
	APJL_0348	YP_001651383.1	*hlpB*	lipoprotein HlpB	41.7	APL_0332	APP7_0337
	APJL_0453	YP_001651480.1	*smpA*	small protein A	37.5	APL_0428	APP7_0452
	APJL_0487	YP_001651512.1	*plpD*	lipoprotein	55.4	APL_0460	APP7_0537
	APJL_0822	YP_001651826.1	*mltA*	murein transglycosylase A	64.1	APL_0816	APP7_0873
	APJL_0922	YP_001651924.1	*hlpA*	outer membrane lipoprotein	53.8	APL_0910	APP7_0970
	APJL_1740	YP_001652736.1	*tolA2*	colicin import membrane protein	55.8	ND^*a*^	ND
III	APJL_0037	YP_001651087.1		hypothetical protein	34.7	APL_0036	APP7_0036
	APJL_0117	YP_001651165.1		hypothetical protein	46.2	APL_0116	APP7_0116
	APJL_0221	YP_001651269.1		putative lipoprotein	48.1	APL_0220	APP7_0222
	APJL_0228	YP_001651276.1		hypothetical protein	35.7	APL_0227	APP7_0229
	APJL_0239	YP_001651287.1		putative lipoprotein	45.2	APL_0234	APP7_0236
	APJL_0605	YP_001651619.1		lipoprotein	43.2	APL_0611	APP7_0657
	APJL_1140	YP_001652140.1		conserved putative lipoprotein	53.6	APL_1121	APP7_1179
	APJL_1310	YP_001652310.1		hypothetical protein	33.9	APL_1297	APP7_1348
	APJL_1318	YP_001652318.1		hypothetical protein	36.3	ND^*a*^	APP7_1356
	APJL_1380	YP_001652380.1		hypothetical protein	87.4	APL_1362	APP7_1413
	APJL_1467	YP_001652463.1		hypothetical protein	42.0	APL_1435	APP7_1497
	APJL_1469	YP_001652465.1		hypothetical protein	62.6	APL_1437	APP7_1495
	APJL_1726	YP_001652722.1		putative ABC transporter periplasmic binding protein	53.9	APL_1694	APP7_1755
	APJL_1919	YP_001652913.1		hypothetical protein	50.9	APL_1875	APP7_1963
	APJL_1942	YP_001652936.1		Zn-dependent protease with chaperone function	51.8	APL_1898	APP7_1985
	APJL_1976	YP_001652968.1		lipoprotein	44.2	APL_1929	APP7_2018
	APJL_1977	YP_001652969.1		hypothetical protein	31.1	ND	APP7_2019
	APJL_2094	YP_001653083.1		hypothetical protein	44.1	APL_2043	APP7_2130

### Immunogenicity and protection of lipoproteins in mice

**Figure 3:**
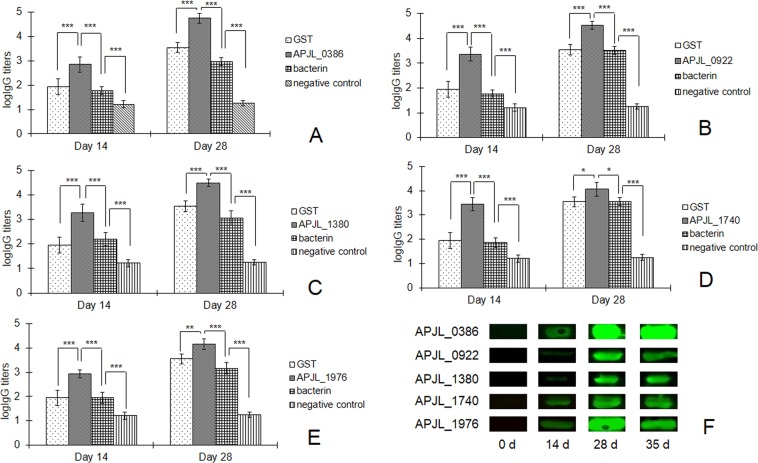
Dynamics of the humoral immune response to lipoproteins in mice determined by ELISA and immunoblotting. Antibodies against lipoproteins APJL_0386 (**A**), APJL_0922 (**B**), APJL_1380 (**C**), APJL_1740 (**D**) and APJL_1976 (**E**) were evaluated at different time points with ELISA. IgG titers were expressed as the logarithm (log10) of the reciprocal of the highest dilution of serum with an OD_630_ value above that of the cutoff value. One-way analysis of variance (ANOVA) was used to compare antibody titers among groups, and the significance level was set at 5%. All ANOVA tests were found to be significant (*P* < 0.001) and were followed up with Student’s *t*-test to compare between any 2 groups. Significant differences between lipoproteins and other groups are outlined with asterisks, *P < 0.05, **P < 0.01, ***P < 0.001. (**F**) For immunoblot analysis, recombinant lipoprotein was separated by SDS-PAGE and transferred onto nitrocellulose membranes. The membranes were cut into small strips, and incubated with pooled antiserum from lipoprotein-immunized mice at days 0, 14, 28 and 35. The strips were incubated with Dylight-800-conjugated goat anti-mouse IgG, and the images were captured with a scanned infrared imaging system.

The protein-specific IgG levels were detected by indirect ELISAs. Specific humoral immune responses of every group were induced 2 weeks after the first immunization, and increased by large margins after booster immunization (Fig. 3). Compared with the GST control, IgG levels of each lipoprotein-immunized group were significantly higher at days 14 and 28 (Fig. 3A–E, *P* < 0.05). Immunization with bacterin induced humoral immune responses against these lipoproteins. The antibody levels in the bacterin-immunized group were lower than those in the lipoprotein-immunized groups (*P* < 0.05), but significantly higher than those in the negative control group (Fig. 3A–E, *P* < 0.001).

Immunoblot analysis was used to confirm the humoral immune response of mice upon vaccination. No signal was observed before immunization, and weak signals were detected 2 weeks after the first immunization (Fig. 3F). Immunoblotting showed strong signals before challenge, and remained stable at 1 week after challenge.

**Figure 4:**
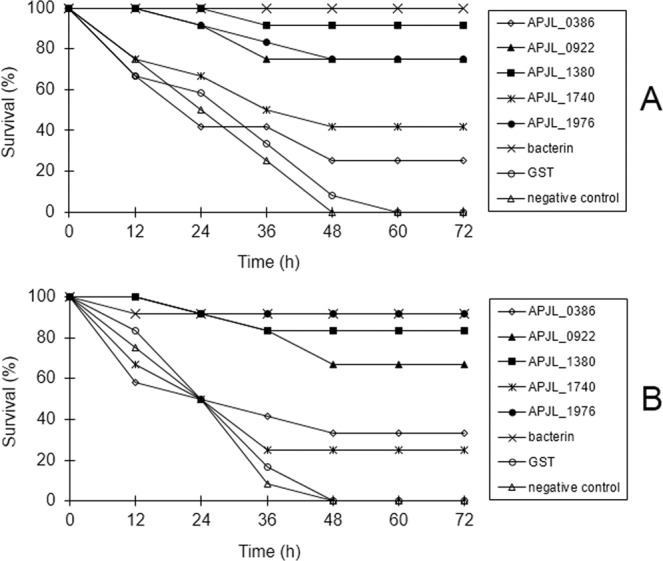
Survival rates of mice in the active immunization (**A**) and passive immunization (**B**) assays. Survival of mice was monitored for 7 days after challenge. Numbers of surviving mice did not change 72 h after infection.

**Figure 5:**
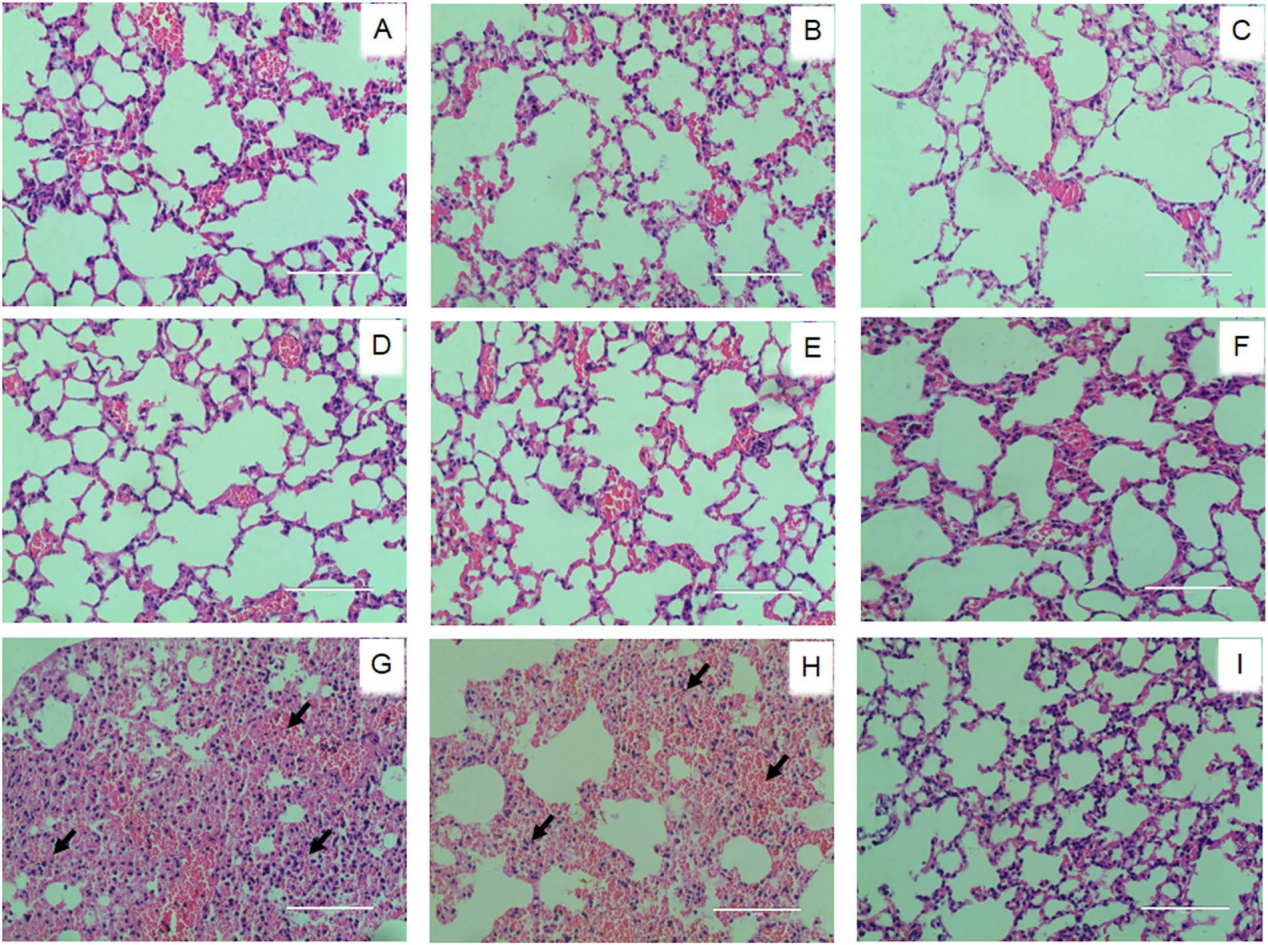
Histopathological evaluation of lung tissues of mice after lethal virulent *A. pleuropneumoniae* serovar 1 challenge. Mice in groups immunized with lipoproteins APJL_0386 (**A**), APJL_0922 (**B**), APJL_1380 (**C**), APJL_1740 (**D**), APJL_1976 (**E**), as well as bacterin (**F)** showed normal lungs under microscopy. Lung tissues from mice in the GST-immunized group (**G**) and negative control group (**H**) exhibited serious inflammatory infiltration, with serous exudates with neutrophils and erythrocytes being observed in many bronchioles and alveoli (black arrows). Lung sections of the healthy mice (**I**) served as a physiological control. The white band at the lower right corner of each picture indicates the scale bar (100 μm).

The survival rates of mice are shown in Fig. 4A, and all mice in the GST and negative control groups were suffered severely from the infection and were euthanized within 48 h after challenge. Although clinical signs were observed in the lipoprotein-immunized groups, animals in these five groups exhibited mild signs and could survive for a long time. Immunization with APJL_1380 conferred 92% protection, and APJL_0922 and APJL_1976 conferred 75% protection from lethal challenge, while the protective efficacy of APJL_0386 and APJL_1740 were 25% and 42%, respectively. Bacterin had a high protective effect and group 7 mice exhibited no mortality within the observation period (Fig. 4A^)^. Histopathological examination showed serious inflammatory infiltration in the lung tissues from the groups that received GST and PBS; many bronchioles and surrounding alveoli were filled with serous exudates with neutrophils and erythrocytes, indicating that the animals had acute and hemorrhagic pneumonia (Fig. 5). Surviving mice in the immunized groups showed normal lung sections, suggesting that they had recovered from acute virulent infection (Fig. 5).

To confirm antibody-mediated protection, mice were passively immunized with hyperimmune serum against these antigens in the active immunization assay. Antisera raised against proteins APJL_1380 and APJL_1976 significantly protected mice from lethal heterologous *A. pleuropneumoniae* challenge, with survival rates of 83% and 92%, respectively (Fig. 4B). The antiserum against protein APJL_0922 conferred 67% protection. Survival rates of mice that received antiserum against APJL_0386 and APJL_1740 were 33% and 25%, respectively. No mice inoculated with antiserum against GST or PBS survived challenge. Antibodies induced by bacterin provided 92% protection in mice. These data indicated that immunization with these lipoproteins, especially APJL_0922, APJL_1380 and APJL_1976, provided considerable cross-serovar protection against *A. pleuropneumoniae* infection, and the protective immunity was at least partially mediated by the antibodies against these proteins.

### Humoral immunity and protective efficacy in pigs

The levels of antibodies against APJL_0922, APJL_1380, and APJL_1976 in groups 1, 2 and 3, respectively, were significantly higher than that in the bacterin and negative control groups before challenge (*P* < 0.001, Table 3), and the bacterin-immunized group showed higher antibodies levels than that of the negative control group (*P* < 0.001, Table 3).

**Table 3:** IgG titers of pigs vaccinated with different immunogens.

Group	Immunogen	IgG titer against APJL_0922 (mean ± SD)^*a*^		IgG titer against APJL_1380 (mean ± SD)		IgG titer against APJL_1976 (mean ± SD)	
		Day 14^*b*^	Day 28	Day 14	Day 28	Day 14	Day 28
1	APJL_0922	2.75 ± 0.13 ^*c,d*^	3.97 ± 0.16^*c,d*^	ND	ND	ND	ND
2	APJL_1380	ND	ND	2.69 ± 0.16^*c,d*^	4.17 ± 0.25^*c,d*^	ND	ND
3	APJL_1976	ND	ND	ND	ND	2.69 ± 0.50^*d,e*^	3.75 ± 0.39^*c,d*^
4	Bacterin	1.78 ± 0.16^*d*^	3.32 ± 0.16^*d*^	1.66 ± 0.13 ^*f*^	2.48 ± 0.16^*d*^	1.72 ± 0.16 ^*f*^	2.36 ± 0.13^*d*^
5	Negative control	1.18 ± 0.16	1.38 ± 0.25	1.30 ± 0.21	1.16 ± 0.22	1.36 ± 0.25	1.32 ± 0.18

^*a*^The solid-phase antigen in ELISAs were prepared as described in the text. The results given are the arithmetic mean log_10_ values of the reciprocal of the highest serum dilution showing an optical density twice as high as the negative control serum.

^*b*^Day 14 means before the second vaccination; Day 28 means before challenge.

Differences in antibodies titers among groups were calculated by one-way ANOVA followed up with Student’s *t*-test to compare between any 2 groups. All ANOVA tests were found to be significant (*P* < 0.001 for ELISA titers against APJL_0922 and APJL_1380; *P* < 0.01 for ELISA titers against APJL_1976).

For Student’s *t*-test between two groups, ^*c*^*P* < 0.001 compared to bacterin, ^*d*^*P* < 0.001 relative to negative control, ^*e*^significantly different from bacterin (*P* < 0.01), ^*f*^significantly different from negative control (*P* < 0.05).

Though all pigs survived from the virulent *A. pleuropneumoniae* challenge, pigs in the negative control group showed significant clinical signs of porcine pleuropneumonia, such as high temperature, decreased appetite, frequent and rough cough, and lethargy, suggesting that the challenge dose used in this trial is high enough to cause typical symptoms in the unimmunized pigs. Both the lipoprotein immunized and the bacterin vaccinated groups individually showed slight to moderate clinical signs at first 48 h post challenge (Table 4). However, the clinical scores were significantly lower compared with the negative control group (*P* < 0.01, Table 4). There was no significant difference between the bacterin vaccinated group and the APJL_1380 vaccinated group (*P* > 0.05), but the clinical scores of APJL_0922 and APJL_1976 vaccinated groups were higher than those of the bacterin vaccinated group (*P* < 0.05, Table 4).

**Table 4:** Protection of pigs vaccinated with lipoproteins against challenge with virulent *A. pleuropneumoniae*.

Group	Immunogen	Arithmetic mean ± SD of clinical signs score^*a*^	No. of pigs with lung lesion/total no.	Arithmetic mean ± SD of pleuritis (%)^*b*^	Arithmetic mean ± SD of lung lesion score^*c*^
1	APJL_0922	1.60 ± 0.55^*d,e*^	3/5	3.80 ± 3.63^*d*^	2.40 ± 2.30^*d*^
2	APJL_1380	0.80 ± 0.84^*d*^	3/5	2.80 ± 2.77^*d*^	1.80 ± 1.79^*d*^
3	APJL_1976	1.60 ± 0.84^*e,f*^	4/5	5.20 ± 3.27^*d*^	3.20 ± 1.92^*d*^
4	Bacterin	0.60 ± 0.55^*d*^	3/5	2.00 ± 1.87^*d*^	1.40 ± 1.34^*d*^
5	Negative control	3.6 ± 0.55	5/5	30.20 ± 7.46	14.40 ± 2.51

^*a*^Clinical signs score of challenged pig was assessed as described by Tumamao *et al*.^46^: 0, no signs; 1, increased respiration; 2, abdominal breathing; 3, cough; 4, dyspnoea and 5, euthanasia due to severe respiratory distress. The highest clinical signs score of each pig was recorded for evaluation.

^*b*^Percentage of pleural surface area exhibiting pleuritis.

^*c*^The lung lesion score was recorded as described previously^47^. A complete lung was divided into 7 lobes and each was arbitrarily allotted a maximum possible lesion score of 5. Lesions of each lobe were assessed and recorded as a fraction of 5, and the lung lesion score was calculated as the sum of the 7 lobes.

Differences in clinical signs, pleuritis and lung lesion scores among groups were analyzed using ANOVA and followed up with Student’s *t*-test to compare between any 2 groups. All ANOVA tests were found to be significant (*P* < 0.001).

For Student’s *t*-test, ^*d*^*P* < 0.001 compared with negative control, ^*e*^ignificant difference relative to bacterin (*P* < 0.05), ^*f*^significant difference relative to negative control (*P* < 0.01).

**Figure 6:**
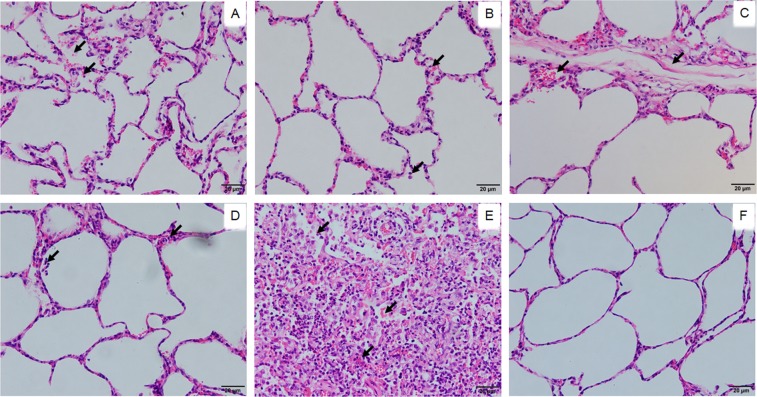
Histopathological examinations of pigs after challenge. Pigs were immunized with lipoproteins and bacterin twice, respectively, and challenged with virulent *A. pleuropneumoniae*. The lower right corner of each picture indicates the scale bar (20 μm). (**A**) APJL_0922, arrows indicate exudations include erythrocytes and alveolar epithelial cells in alveoli. (**B**) APJL_1380, arrows indicate slight congestion of alveolar walls and exfoliated alveolar epithelial cells. (**C**) APJL_1976, congestion and thickness of pulmonary interlobular septa. (**D**) Bacterin, swelling of alveolar walls. (**E**) The negative control, severe inflammatory infiltration in alveoli, with neutrophils, alveolar epithelia cells and erythrocytes. (**F**) Health lung tissue shows normally inflated alveoli.

All unvaccinated pigs showed multiple lung lesions and adhesive pleuritis during postmortem examination. In contrast, there’s no obvious lung lesions and pleuritis in the bacterin-immunized group, and only minor lung lesions and exudation were observed in the protein immunized groups (Table 4). The histopathological analysis result is consistent with that of the postmortem examination. Lung sections from the lipoprotein-immunized groups showed normal to mild infiltrates, less serious than those of unvaccinated pigs, which displayed severe exudation and hemorrhagic pneumonia (Fig. 6).

## Discussion

Porcine pleuropneumonia caused by *A. pleuropneumoniae* is a major problem in the pig industry. Vaccination is considered to be one of the most promising strategies for control of the disease. However, traditional vaccines on the market against *A. pleuropneumoniae* show a varying degree of defects in terms of cross-protection and protection against morbidity and mortality^9,10^. Development of novel types of vaccines and identification of candidate vaccine antigens are important approaches to overcome the deficiencies of current vaccines, and have received much attention in recent years^10^. Immunogenic proteins from the cell surface and outer membrane of *A. pleuropneumoniae* have been identified by phage display, immunoproteomic analysis or bioinformatic prediction^12,20,21^. In addition, antigens expressed during *A. pleuropneumoniae* infection were found using the *in vivo*-induced antigen technology^22^. The protective immunity of some identified proteins was investigated, although only a few of the tested proteins provided partial protection, and most of them induced considerable seroconversion in inoculated animals, suggesting that they are potential candidates for subunit vaccines^13,20,21^. These results demonstrate that high-throughput screening and bioinformatics technologies are powerful tools in identifying novel vaccine antigens. We targeted bacterial lipoproteins in the present study to screen for protective antigens for vaccine development.

Although recombinant proteins in an insoluble form might be efficacious antigens, in this study, we focused on proteins expressed in soluble form, because of the importance of protein conformation in eliciting protective immune responses, and of the fact that soluble recombinant proteins can be more easily purified by affinity chromatography. For this purpose, hydrophobic signal peptides, which are often found embedded in membranes and are difficult to fold correctly when overexpressed in *E. coli*^23^, were removed from these proteins by expressing truncated versions of the genes. Finally, 37 of 47 expressed recombinant proteins were detected in the supernatant of *E. coli* lysate (Table S2). To exploit serovar-cross reactive/protective vaccine candidates from these soluble lipoproteins, which are encoded by an *A. pleuropneumoniae* serovar 3 strain, polyclonal antibodies against *A. pleuropneumoniae* serovar 7 was used as the primary antibody in the western blot analysis. In addition, animals immunized with these selected lipoproteins were challenged by a virulent heterologous *A. pleuropneumoniae* strain (4074, serovar 1).

Considering that mouse could be an alternative in the study of *A. pleuropneumoniae* infection in the laboratory^24^, and the mouse model has been used to evaluate *A. pleuropneumoniae* vaccine candidates successfully in many previous studies^25,26^, the immunity potential of *A. pleuropneumoniae* lipoproteins was tested preliminarily in mice. Results indicate that these selected lipoproteins were able to stimulated protective immunity in mice against challenged by lethal virulent *pleuropneumoniae* serovar 1. Then the protective efficacy of three selected lipoproteins were verified in the natural host, the pigs in this study. Pigs immunized with lipoproteins elicited considerable antibodies against target proteins before challenge (Table 3), confirming that recombinant lipoproteins produced in *E. coli* are immunogenic. Pigs vaccinated with lipoproteins, showed few clinical symptoms or lung lesions after challenge with virulent heterologous serovar 1 (Table 4). In addition, the histological examinations confirm pigs in the unvaccinated control group infected with *A. pleuropneumoniae* serovar 1 suffered from pleuritis and hemorrhagic pneumonia, whereas lung sections from lipoprotein-immunized groups showed less pathological changes than those of unvaccinated groups (Fig. 6). Taken together, results indicate that these three lipoproteins provide effective protection for pigs against virulent *A. pleuropneumoniae* challenge. Our present data showed a picture of immunity potential of *A. pleuropneumoniae* lipoproteins.

An important observation of this study was that several antigens have been reported to be immunogenic or protective in *A. pleuropneumoniae*. The PotD protein, which is reported to be a potential vaccine candidate against *Streptococcus pneumoniae* infection^27^, was recently shown to be involved in the growth, stress tolerance and virulence of *A. pleuropneumoniae*^28^. The outer membrane, lipoprotein-trafficking protein LolB^29^, delays the development of disease in pigs upon challenge with virulent *A. pleuropneumoniae*^20^. Although PlpB is a potential target of subunit vaccine against *Pasteurella multocida*^30^, it was not protective against *A. pleuropneumoniae*, probably because the recombinant *A. pleuropneumoniae* PlpB protein was not folded into a soluble conformation when expressed in *E. coli*^31^. The *A. pleuropneumoniae* HgbA protein is homologous to the *Haemophilus influenzae* periplasmic HbpA protein, which has been implicated in the uptake of heme and glutathione, and associated with bacterial virulence^32,33^. Immunization with a single HbpA protein provided partial protection for mice against challenge with virulent *A. pleuropneumoniae*^21^. In the present study, all four of these proteins were recognized by the rabbit anti-*A. pleuropneumoniae* serum by western blotting (Fig. 3). Immunization of mice with PotD elicited a high level of humoral immune response, and conferred partial protection on mice in our vaccine trials. These data suggest that our experimental approach based on bioinformatics and immunological testing is capable of selecting effective candidate antigens.

The list of candidate lipoproteins in Table 2 includes 13 soluble expressed proteins, which have been functionally annotated according to the sequence similarities with other bacterial species^34^. In addition to the reported proteins described above, five antigens exhibited strong signals in western blotting analysis in our study: SlyB (APJL_0038), PlpD (APJL_0487), TadD (APJL_0542), HlpA (APJL_0922) and TolA (APJL_1740). Involvement of these factors in cellular processes and infection with *A. pleuropneumoniae* and some other pathogens have been mentioned before. In *Burkholderia multivorans*, the outer membrane lipoprotein SlyB has been shown to contribute to membrane integrity^35^. PlpD was found to be homologous with the *E. coli* BamE, which is the core component of β-barrel assembly machine (Bam), and is reported to regulate outer membrane integrity and cell viability^36^. TadD of the tight adherence (tad) locus is up-regulated when *A. pleuropneumoniae* is cultured in biofilm-formation medium, emphasizing the possible importance of TadD for biofilm formation^37^. APJL_0922 encodes a protein with homology to d-methionine-binding lipoprotein MetQ of other bacteria. Previous studies have suggested that MetQ is involved in methionine transport, cell adherence, intracellular survival in *Neisseria gonorrhoeae*, and more importantly, the surface-located MetQ protein is a promising vaccine candidate that elicits bactericidal and functional blocking antibodies^38^. The *A. pleuropneumoniae* TolA protein is homologous to the colicin transporter of *Neisseria sicca*, and contains 15 copies of EAEAKAKA in the intragenic tandem repeat (TR) region at the *N* terminus, and is implicated in *E. coli* colicin uptake, filamentous phage infection, and detergent tolerance^39^. The TR region of TolA was truncated in the present study, so as to increase the production and solubility of the immunogenic *C* terminus of TolA. Two proteins, HlpA and TolA, which showed strong positive signals in our western blotting analysis, were used for animal experiments. These two proteins, especially HlpA, provided significant protection in our mouse model. Our results support the findings that at least some immunoreactive lipoproteins are protective vaccine candidates.

A particularly interesting finding is the identification of 23 lipoproteins that so far have only been annotated as hypothetical. It is worth noting that a number of hypothetical proteins (APJL_0221, APJL_1318, APJL_1380, APJL_1469, APJL_1726 and APJL_1976) yielded clearly positive reactions with rabbit anti-*A. pleuropneumoniae* serum in western blotting. Two hypothetical lipoproteins, APJL_1380 and APJL_1976, were selected for further investigation. On the basis of *in silico* analysis, APJL_1380 is homologous to *E. coli* outer membrane, penicillin-binding protein activator LpoA, which is reported to stimulate the transpeptidase activity of peptidoglycan synthase PBP1A and thus regulate peptidoglycan synthesis^40,41^. However, the roles of LpoA in immunological protection and pathogenesis have not been addressed before. Here, our data showed that APJL_1380 elicited effective protection in animals in both active and passive immunization assays. To our knowledge, this is the first report of the vaccine potential of the LpoA lipoprotein homolog from γ-proteobacteria. The hypothetical protein APJL_1976 is homologous to the *H. influenzae* NlpD. NlpD has been identified as being cell-surface located and is important for membrane stability of *H. influenzae*^42^, and it is reported to be essential for pathogenicity of *Yersinia pestis*^43^. Immunization with APJL_1976 could induce high IgG titer and confer partial protection against infection with virulent *A. pleuropneumoniae*. These results suggest that many immunogenic antigens are still undiscovered in *A. pleuropneumoniae*. Further investigation of the biological roles of these hypothetical proteins may provide insight into the pathogenesis of *A. pleuropneumoniae*.

In summary, this work represents a systematic analysis of immunological properties of *A. pleuropneumoniae* lipoproteins. The data present here provide important information for exploration of novel subunit vaccines. In addition, they also provide clues for further development of new diagnostic assays for assessment of *A. pleuropneumoniae* infection, and for investigation of the molecular mechanisms of *A. pleuropneumoniae* pathogenicity. We know that one immunogenic protein often elicits limited protection against bacterial infection; thus, bacterial subunit vaccines contain a number of protective antigens. However, a recent study indicated that administration of certain combinations of *A. pleuropneumoniae* protective components results in more serious lesions than those induced by individual immunogens upon challenge^14^. Therefore, we are currently investigating different vaccine formulations based on the *A. pleuropneumoniae* Apx toxins, by the addition of these newly discovered vaccine candidates.

## Materials and Methods

### Bacterial strains, plasmids, primers and growth conditions

The bacterial strains and plasmids used in this work are listed in Table 1. The primers (Table S1) were designed according to the coding sequences of predicted lipoprotein mature peptides in the *A. pleuropneumoniae* JL03 (serovar 3) genome and synthesized by Sangon Biotech (Shanghai) Co. Ltd. (Shanghai, China). *A. pleuropneumoniae* strains were incubated in tryptic soy broth (TSB) or on tryptic soy agar (TSA) (Becton, Dickinson & Co., Franklin Lakes, NJ, USA) with 10% calf serum and 10 μg/ml nicotinamide adenine dinucleotide (Sigma–Aldrich, St. Louis, MO, USA). *E. coli* strains were cultured in Luria–Bertani broth or agar, supplemented with 100 μg/ml ampicillin (Sigma-Aldrich).

### Generation of recombinant lipoproteins

*A. pleuropneumoniae* JL03 was cultured on TSA plates, then single colonies were picked and incubated in TSB medium. Genomic DNA was extracted from *A. pleuropneumoniae* culture using a genomic DNA mini preparation kit (Boyue Biotech Co. Ltd., Wuhan, China). The construction of the expression plasmid and the generation of recombinant lipoprotein were as follows. The DNA coding sequence was amplified from the genomic DNA by polymerase chain reaction (PCR) with specific primers (Table S1), and ligated into the A/T cloning vector pMD18-T (Takara, Dalian, China) and sequenced in both directions. Then it was cut from pMD18-T vector by restriction enzyme digestion and ligated into the prokaryotic expression vector pGEX-KG^44^, so as to generate the expression plasmid, which was then transformed into *E. coli* BL21(DE3). Recombinant protein was produced by isopropyl-β-D-thiogalactoside (1 mM, Sigma-Aldrich) induction of *E. coli* containing the expression plasmid. The recombinant glutathione transferase (GST)-fusion protein in the supernatant of *E. coli* cell lysate was purified with a glutathione–Sepharose 4B affinity chromatography column (Amersham Biosciences, Little Chalfont, UK).

### Western blotting

For testing of the immunoreactivity of recombinant lipoproteins, purified recombinant lipoproteins were separated on sodium dodecyl sulfate-polyacrylamide gel electrophoresis (SDS-PAGE). Proteins on the gel were transferred onto nitrocellulose membranes under electronic field and capillary penetration. The nitrocellulose membranes were blocked using Tris-buffered saline with 0.5% Tween-20 (TBST) and 5% skimmed milk at room temperature for 1 h. After three washes with TBST, the membranes were incubated with rabbit polyclonal antibodies against *A. pleuropneumoniae* serovar 7 strain WF83^45^ (1:400 diluted with TBST) for 30 min at room temperature. The membranes were washed four times with TBST, and then incubated with Dylight-800-conjugated goat anti-rabbit IgG (Abbkine Scientific Co. Ltd., Wuhan, China; 1:5000 diluted with TBST) for 40 min at room temperature. After four washes with TBST and three washes with TBS, images were captured using a scanned infrared imaging system (Odyssey; LICOR, Lincoln, NE, USA). Western blotting was also used to evaluate the lipoprotein-specific antibodies in mice, pooled serum from each lipoprotein-immunized group was used as the primary antibody and Dylight-800-conjugated goat anti-mouse IgG was used as the secondary antibody (Abbkine). The western blotting analysis was proceeded as described above.

### Immunization assays in mice

#### Active immunization assay

Ninety-six 6-week-old female BALB/c mice were purchased from the Center for Disease Control of Hubei Province (Wuhan, China) and randomly assigned into eight groups, each of 12 animals. The animal immunization and infection experiments were approved by the Animal Ethics Committee at the Central China Normal University, and carried out under the Guidelines for the Care and Use of Laboratory Animals provided by this Committee (No. SYXK 2015-0052). Mice were housed in sterile isolators and fed with sterile food and water. Mice in groups 1–5 were immunized with five selected recombinant lipoproteins (APJL_0386, APJL_0922, APJL_1380, APJL_1740 or APJL_1976), and group 6 was immunized with recombinant GST and used as a tag protein control. Proteins (800 μg/ml) were emulsified with an equal volume of complete Freund’s adjuvant for the first immunization, and emulsified with an equal volume of incomplete Freund’s adjuvant for the booster immunization. Group 7 was immunized with a commercial trivalent inactivated vaccine (containing *A. pleuropneumoniae* serovars 1, 2 and 7; Wuhan Keqian Biotech, China). Group 8, injected with phosphate-buffered saline (PBS), was used as a negative control. Mice were injected intraperitoneally with 0.2 ml immunogen and boosted 14 d after the first immunization. To verify the possible cross-serovar protection potential of target lipoproteins, in this study, the infection assays were performed using an *A. pleuropneumoniae* 4074 (serovar 1) instead of *A. pleuropneumoniae* JL03 (serovar 3). Two weeks after booster immunization, all mice were challenged intraperitoneally with heterologous and virulent *A. pleuropneumoniae* strain 4074 (serovar 1, 5.0^ × ^10^6^ CFU/each) in 0.5 ml TSB. Mice were monitored for 7 d after challenge; clinical signs were recorded, and dying mice that showed obvious dyspnea and lethargy were euthanized. Surviving mice were euthanized after the observation period. Lung tissues collected from dying and surviving mice were fixed in formalin. Thin sections (5 µm) sections were prepared using hematoxylin–eosin staining and analyzed by microscopy.

#### Passive immunization and challenge

For passive immunization, serum samples in the active immunization assay as mentioned above were collected before challenge and pooled. Ninety-six 6-week-old female BALB/c mice were purchased from Hubei CDC and divided into eight groups randomly. Each mouse in groups 1–5 was injected intraperitoneally with 50 μl pooled antiserum against recombinant lipoproteins APJL_0386, APJL_0922, APJL_1380, APJL_1740 and APJL_1976, and mice in groups 6 and 7 were injected with antiserum against GST and bacterin, respectively. Group 8 was inoculated with serum from the negative control group in the active immunization assay. Three hours later, mice were challenged with 5.0 × 10^6^ CFU *A. pleuropneumoniae* strain 4074. Survival rates of mice were monitored for 7 d after challenge.

#### Vaccination and challenge in pigs

According to the results from mice Immuno-protection assays, three lipoproteins APJL_0922, APJL_1380, and APJL_1976 showed better protection were further investigated against virulent *A. pleuropneumoniae* challenge in pigs. Twenty-five pigs were purchased from *A. pleuropneumoniae*-free herd and randomly divided into 5 groups of 5 pigs each. Groups 1–3 were vaccinated intramuscularly (i.m.) twice with 2 ml of recombinant lipoproteins APJL_0922, APJL_1380, and APJL_1976, respectively, at an interval of 2 weeks. Immunogens were prepared in the same manner as described in the mouse immunization assay. Group 4 was inoculated i.m. twice with 2 ml of the inactivated vaccine. Group 5 was injected i.m. twice with 2 ml of PBS as a control. Two weeks after the second immunization, the animals were challenged intratracheally with 5 × 10^7^ CFU of *A. pleuropneumoniae* 4074 in 2 ml PBS. Clinical symptoms were recorded according to the methods described by Tumamao *et al*.^46^. Pigs displaying serious respiratory dyspnea were immediately euthanized, and the remaining pigs were euthanized 7 days post-challenge for postmortem examination, lung lesion scores were recorded as described previously^47^. For histological examination, lung samples were fixed in formalin, and sections were prepared as described above.

#### Evaluation of humoral immune responses

Blood samples were collected from animals (the tail vein for mice and the front cavity vein of pigs) 1 d before each immunization and before challenge. The levels of serum antibodies against target proteins were measured as described previously with minor modifications^18^. Enzyme-linked immunosorbent assay (ELISA) plates were coated with appropriate amounts of purified proteins (0.2–0.3 μg/well for each protein), and blocked with PBST (PBS with 0.05% Tween 20) plus 5% skimmed milk for 1 h. Serum samples were serially diluted and added to coated ELISA plates, and incubated at 37 °C for 30 min. After four washes, 100 μl horseradish peroxidase (HRP)-labeled goat anti-mouse (or anti-porcine) IgG (Southern Biotechnology Associates, Birmingham, AL, USA) diluted 1:5000 in PBST was added to each well, and the plate was incubated at 37 °C for 30 min. The plate was washed five times, then color was developed with a 3,3′,5,5′-tetramethylbenzidine color development kit (Tiangen Biotech Co. Ltd., Beijing, China). The catalytic reaction was stopped with 50 μl 1% SDS. The optical density was read at 630 nm (OD_630_) in an ELISA microplate reader (PowerWave XS; Bio-Tek, Winooski, VT, USA). The antibody titers were expressed as the reciprocal of the highest dilution giving an OD_630_ value above the cutoff value.

## Supplementary information

Table S1 + Table S2.
