# Supplementary material for: Genome-wide screening of lipoproteins in Actinobacillus pleuropneumoniae identifies three antigens that confer protection against virulent challenge
Source: Sci Rep. 2020 Feb 11;10:2343. doi: 10.1038/s41598-020-58968-7 (PMC7012816; doi:10.1038/s41598-020-58968-7)
Supplement: Supplementary file 1 — Table S1 + Table S2. [file 41598_2020_58968_MOESM1_ESM.pdf]

Genome-wide screening of lipoproteins in *Actinobacillus pleuropneumoniae* identifies three antigens that confer protection against virulent challenge

Yurou Cao, Lulu Gao, Li Zhang, Lixiang Zhou, Jihong Yang, Lingfu Deng,

Jin Zhao, Chao Qi*, Jinlin Liu*

Table S1. Primers used in this study.

No. of lipoprotein	Gene locus_tag	Forward primer	Reverse primer	Size of target genes
1	APJL_0037	TTGGATCCTGTACTCAAGATTTCTATGCC	GGAAGCTTTTATTTGAATTCATAACGACG	228
2	APJL_0038	GTGGATCCTGTGCAAATACCGATATTTAC	TGAAGCTTTTACAGCACAGACACGTTC	441
3	APJL_0100	GGAGATCTTGTTTATCATCAGCAGCA	GGTCTAGATTATTTCGCTTCTGCTTTTG	1458
4	APJL_0117	GGAGATCTTGTTCGAGTAAAAGTAGTTG	GGAAGCTTTTATTTAGCCGATTTCTGT	528
5	APJL_0126	TTGGATCCTGCGGCAGTAATCCGCAAAG	GGAAGCTTTCATCGGTTACCACGTAA	318
6	APJL_0157	TTGGATCCTGTAACAAAGCTCCGGAG	GGAAGCTTGCTCGACATTTTGGTTTCA	978
7	APJL_0221	TTGGATCCTGTGCAACTGAAGGTTCTA	GGAAGCTTTTATTTAGCAGGAGTCCAAT	621
8	APJL_0223	TTGGATCCTGCGCTCAACAGCAGAAAC	GGAAGCTTTCCATATAAAGAATGGCGA	612
9	APJL_0228	TTGGATCCTGTTCCGCACCCGAGCAACA	GGAAGCTTTTACCAATGGTGTCTATGAT	249
10	APJL_0239	TTGGATCCTGCCAATCGCAATCAAACG	GGAAGCTTTTATTTAAACTGGTGAATTGC	528
11	APJL_0250	TTGGATCCTGTTCAGGTGGTAAAGGTG	GGAAGCTTCTATTTCTTCTCGACTTGAC	1653
12	APJL_0265	GGGGATCCTGTTCAACCACAAAATTAAGTG	GGAAGCTTCTATCTTCTATATTTACCCG	1341
13	APJL_0347	TTGGATCCTGCGATAAACCGGCAAATAAG	GGAAGCTTACCGTATTTGGTTGCTAATTC	699
14	APJL_0348	TTAGATCTTGTAACAAAGCGGATCCTG	GGAAGCTTTTTCGCTTTTAAATCGGCT	423
15	APJL_0373	TTGGATCCTGTGCCGTCACGCCGCAG	GGAAGCTTTTACCAACAGGCATAACAGT	333
16	APJL_0386	GGGGATCCTGTAATGAAGAAAAGCCAAA	GGAAGCTTTTATTTCGCCGCTTTTAACTC	1026
17	APJL_0410	TTGGATCCTGCGCTCATCACGATACGA	TTAAGCTTTTATTTGCCGTCCCAAGCC	759
18	APJL_0453	TGGGATCCTGTTCTACCGTAAAAAAAGTC	GGAAGCTTTTGCGCTTCATTCGTAATAG	291
19	APJL_0487	TTGGATCCTGCGGTAATTTAAGTAACG	GGTCTAGATTATTTGCCGGAGTTATATG	789
20	APJL_0542	TTGGATCCTGCTCTAACTTGACGGCAA	GGAAGCTTTTAACGTCCCACTTTAGGTG	693
21	APJL_0586	TTGGATCCTGTGCTATTTTCGGTCCATC	GGAAGCTTTGGTTTTATTGAAAGGTAAG	273
22	APJL_0605	GGGGATCCTGTTCAAATGATATTAGCG	TTAAGCTTTTATAAACGGCGTGCTTGCC	456
23	APJL_0633	GGGGATCCTGTTCAAATAAAAATAGTACG	TTAAGCTTTTACCGCCCCACGCTTTCTA	1035
24	APJL_0780	TTGGATCCTGCCAATCGGTATTAAACGA	TGAAGCTTCTAAAACGCCCATTGATCAAC	579
25	APJL_0822	TTGGATCCTGTTCTTCGGATAGAGTAAG	GGAAGCTTTTAGTTTAATACCCAAACACGT	1035
26	APJL_0885	TTGGATCCTGCGGTTGGCACTTCAAAAAC	GGTCTAGATTCTTTATGTAAACCGGCCA	450
27	APJL_0921	TTGGATCCTGTAAAGAAGAGAAAGCGG	GGAAGCTTTTACCAGCCTTTTACAATACC	762
28	APJL_0922	TGGGATCCTGTAAAGAAGAGAAGAAAGC	GGAAGCTTTTACCAACCTTTTATCACAC	76
29	APJL_0931	TTGGATCCTGTAAATCTAGCCATGGTC	GGTCTAGACTATTTTTCAGTTTTCACTGC	1197
30	APJL_0973	ACGGATCCTGTTCATTTGGTGGATTTAAACC	AGTCTAGATTATTTCCCCCAAGGTTTC	309
31	APJL_1078	TTGGATCCTGCTCAACGAAAAACGAAAC	GGAAGCTTTTATTGTACTTTTTCTACGC	312
32	APJL_1140	TTGGATCCTGTTCAAGTGCGAATAAAG	GGAAGCTTGAATTGTTCGCTATATTCAG	720
33	APJL_1172	TTGGATCCTGTTCAACCGCTAATGATGT	GGAAGCTTTTTATTTAACTTTGCAGTTGC	471
34	APJL_1284	TTGGATCCTGTTCTTCTCAGCTTCCAAC	TTAAGCTTCTAGCGCAGTGCCGCCGCT	486
35	APJL_1310	TTGGATCCTGTTCCTCTCATCAAAAGG	GGTCTAGATTAGTAGGAATGATGGCGAG	216
36	APJL_1318	TTGGATCCTGTTCGTCACCAAATGATC	GGTCTAGATTATTTGGTTTTAGTGCTTTTC	279
37	APJL_1380	TTGGATCCTGTACCGGTACAAGTTTTTTTG	GGAAGCTTTTAGTTAGCGTTTTCCACTG	1677

38	APJL_1383	TTAGATCTTGTATCGCTACTGCGGTTG	GGAAGCTTTTATTGTACGTAATTAATCAC	522
39	APJL_1429	TTGGATCCTGTAAGCCGTTGGAAGCGC	GGAAGCTTTTATTTGGCTTTACAGTCGC	387
40	APJL_1467	TTGGATCCTGTGATCAAGCTAACCAAG	GGAAGCTTTTATTTAGTTTCAACTGCTTG	459
41	APJL_1469	GGGGATCCTGTGATGATAAAAATACGCAG	GGAAGCTTTTACTCATTCTCGGCTTGG	954
42	APJL_1502	TTGGATCCTGTACTTCTTCCGTGCCGA	GGAAGCTTTTAACTTTCTCGACAAGCTAAC	411
43	APJL_1607	TTGGATCCTGTTCCTCTGCTCCGCAGC	GGTCTAGATTACAGTAATAGCATCGAACC	507
44	APJL_1615	TTAGATCTTGCTCAAGCCTCCCAACTTC	GGAAGCTTTTAATAGGCACGAACGGCAT	1125
45	APJL_1666	GGGGATCCTGTCATTCATTAAACAAAACC	GGAAGCTTTTATTTAACTTGGCTACCG	144
46	APJL_1726	TTGGATCCTGCGATAATGCTAACAATGC	TTAAGCTTTTATTGCGCAAGTAACTCCG	771
47	APJL_1740	TTGGATCCGCGCGTATTGCACAAAAGA	GGAAGCTTTTTTTTGATTTCACCACGAG	840
48	APJL_1777	TTGGATCCTGCGGCAGTAGTTCTCCTA	GGAAGCTTTCGAATGTGTAAATATTTCTCG	1041
49	APJL_1783	TTAGATCTTGTAGTGGCGGCGGCTCAT	GGAAGCTTTGCTTCAGTAGCAGTAGCTC	1038
50	APJL_1919	TTAGATCTTGCGGCAGTCATAAGGATAC	GGAAGCTTTTAGTAAGAATATACTTCGTT	654
51	APJL_1942	TTGGATCCTGCGTCAGTACTCAAAGTAT	TTGGATCCTGCGTCAGTACTCAAAGTAT	711
52	APJL_1960	TTGGATCCTGTAGTACTACCGGTTATC	TTAAGCTTTTTTGCCGATTGCGGGAAG	1050
53	APJL_1965	TTGGATCCTGTTCGTCGTCAATCGATCC	GGAAGCTTTTCCTCTTCGGAAAGCTGTT	699
54	APJL_1976	TTGGATCCTGTTCGTCTTCTTCGTCTTC	GGAAGCTTTTATCTTACACGCAGTATTTG	516
55	APJL_1977	TTGGATCCTGCGGAGCAAGTGAGAACG	GGAAGCTTTTATTTAGTCATTGAAGCCG	150
56	APJL_2004	TTAGATCTTGCTCGTCGGACAATAAAGG	TTAAGCTTAGGATCGGCATTTGTGCAAG	1185
57	APJL_2060	TTGGATCCTGCGATGATAAAAATGCGG	GGAAGCTTTTAGTCAGCAAGTTTTGTAC	1575
58	APJL_2094	GGGGATCCTGTTCTTATAACAACTCAACT	GGAAGCTTTTATTTGTTCGGACGAACAC	483

Table S2. Expression and western blot analyses of recombinant lipoproteins [a]

No. of lipoprotein	Gene locus_tag	Gene name in JL03	Protein_id	Protein solubility [b]	Protein immuno-reactivity [c]	Annotation of target protein in JL03	Homolog of target protein in L20	Homolog of target protein in AP76
1	APJL_0037		YP_001651087.1	soluble	weak positive	hypothetical protein	APL_0036	APP7_0036
2	APJL_0038	*slyB*	YP_001651088.1	soluble	positive	outer membrane lipoprotein	APL_0037	APP7_0037
3	APJL_0100	*nrfA*	YP_001651150.1	insoluble	NT [d]	cytochrome c nitrite reductase subunit c552	APL_0100	APP7_0099
4	APJL_0117		YP_001651165.1	soluble	weak positive	hypothetical protein domain amino terminal to FKBP-type peptidyl-prolyl isomerase	APL_0116	APP7_0116
5	APJL_0126		YP_001651174.1	soluble	negative	Putative periplasmic lipoprotein	APL_0125	APP7_0127
6	APJL_0157	*apbE*	YP_001651205.1	soluble	weak positive	thiamine biosynthesis lipoprotein	APL_0156	APP7_0158
7	APJL_0221		YP_001651269.1	soluble	positive	putative lipoprotein	APL_0220	APP7_0222
8	APJL_0223		YP_001651271.1	insoluble	NT	hypothetical protein	APL_0222	APP7_0224
9	APJL_0228		YP_001651276.1	soluble	weak positive	hypothetical protein	APL_0227	APP7_0229
10	APJL_0239		YP_001651287.1	soluble	weak positive	putative lipoprotein	APL_0234	APP7_0236
11	APJL_0250	*tbpB2*	YP_001651298.1	insoluble	NT	transferrin-binding protein 2	APL_0245	APP7_0246
12	APJL_0265	*tolC*	YP_001651308.1	insoluble	NT	Outer membrane protein	APL_0257	APP7_0259
13	APJL_0347		YP_001651382.1	soluble	negative	putative lipoprotein	APL_0331	APP7_0336
14	APJL_0348	*hlpB*	YP_001651383.1	soluble	positive	lipoprotein HlpB	APL_0332	APP7_0337
15	APJL_0373		YP_001651408.1	unexpressed	NT	hypothetical protein	APL_0356	APP7_0378
16	APJL_0386	*potD*	YP_001651421.1	soluble	positive	spermidine/putrescine ABC transporter periplasmic substrate-binding protein	APL_0368	APP7_0390
17	APJL_0410	*ompP4*	YP_001651443.1	unexpressed	NT	lipoprotein E	APL_0389	APP7_0413
18	APJL_0453	*smpA*	YP_001651480.1	soluble	weak positive	small protein A	APL_0428	APP7_0452
19	APJL_0487	*plpD*	YP_001651512.1	soluble	positive	Peptidoglycan binding domains similar to the C-terminal domain of outer-membrane protein OmpA	APL_0460	APP7_0537
20	APJL_0542	*tadD*	YP_001651561.1	soluble	positive	Flp pilus assembly protein TadD	APL_0549	APP7_0590
21	APJL_0586		YP_001651600.1	unexpressed	NT	hypothetical protein	ND [e]	ND
22	APJL_0605		YP_001651619.1	soluble	weak positive	lipoprotein, NlpC/P60 family	APL_0611	APP7_0657
23	APJL_0633		YP_001651647.1	insoluble	NT	membrane-bound lytic murein transglycosylase B	APL_0642	APP7_0683

24	APJL_0780	*lolB*	YP_001651787.1	soluble	positive	Outer membrane lipoprotein LolB	APL_0777	APP7_0838
25	APJL_0822	*mltA*	YP_001651826.1	soluble	positive	murein transglycosylase A	APL_0816	APP7_0873
26	APJL_0885	*lptE*	YP_001651887.1	soluble	negative	rare lipoprotein B	APL_0873	APP7_0932
27	APJL_0921	*plpB*	YP_001651923.1	soluble	positive	outer membrane lipoprotein 2	APL_0909	APP7_0969
28	APJL_0922	*hlpA*	YP_001651924.1	soluble	positive	outer membrane lipoprotein	APL_0910	APP7_0970
29	APJL_0931		YP_001651933.1	unexpressed	NT	hypothetical protein	APL_0920	APP7_0979
30	APJL_0973		YP_001651975.1	unexpressed	NT	hypothetical protein	ND	ND
31	APJL_1078		YP_001652078.1	soluble	negative	hypothetical protein	APL_1062	APP7_1120
32	APJL_1140		YP_001652140.1	soluble	positive	putative lipoprotein	APL_1121	APP7_1179
33	APJL_1172		YP_001652172.1	unexpressed	NT	hypothetical protein	APL_1152	APP7_1211
34	APJL_1284	*pilF*	YP_001652284.1	unexpressed	NT	putative fimbrial biogenesis and twitching motility protein PilF-like protein	APL_1273	APP7_1323
35	APJL_1310		YP_001652310.1	soluble	positive	hypothetical protein	APL_1297	APP7_1348
36	APJL_1318		YP_001652318.1	soluble	positive	hypothetical protein	ND	APP7_1356
37	APJL_1380		YP_001652380.1	soluble	positive	hypothetical protein	APL_1362	APP7_1413
38	APJL_1383		YP_001652383.1	unexpressed	NT	hypothetical protein	APL_1365	APP7_1416
39	APJL_1429		YP_001652427.1	unexpressed	NT	hypothetical protein	APL_1404	APP7_1535
40	APJL_1467		YP_001652463.1	soluble	positive	hypothetical protein	APL_1435	APP7_1497
41	APJL_1469		YP_001652465.1	soluble	positive	hypothetical protein	APL_1437	APP7_1495
42	APJL_1502		YP_001652498.1	insoluble	NT	hypothetical protein	APL_1477	APP7_1485
43	APJL_1607		YP_001652603.1	insoluble	NT	hypothetical protein	APL_1575	APP7_1637
44	APJL_1615	*cpxD*	YP_001652611.1	insoluble	NT	HexD, capsule biosynthetic locus protein	APL_1582	APP7_1644
45	APJL_1666		YP_001652662.1	soluble	negative	hypothetical protein	APL_1633	APP7_1695
46	APJL_1726		YP_001652722.1	soluble	positive	putative ABC transporter periplasmic binding protein	APL_1694	APP7_1755
47	APJL_1740	*tolA2*	YP_001652736.1	soluble	positive	colicin import membrane protein	ND	ND
48	APJL_1777	*mltC*	YP_001652771.1	insoluble	NT	murein transglycosylase C	APL_1741	APP7_1802
49	APJL_1783	*omlA*	YP_001652777.1	unexpressed	NT	outer membrane lipoprotein A	APL_1748	APP7_1833
50	APJL_1919		YP_001652913.1	soluble	positive	hypothetical protein	APL_1875	APP7_1963
51	APJL_1942		YP_001652936.1	soluble	weak positive	Zn-dependent protease with chaperone function	APL_1898	APP7_1985
52	APJL_1960	*pepO*	YP_001652952.1	insoluble	NT	Predicted metalloendopeptidase	APL_1913	APP7_2002
53	APJL_1965	*vacJ*	YP_001652957.1	unexpressed	NT	VacJ lipoprotein	APL_1918	APP7_2007

54	APJL_1976		YP_001652968.1	soluble	positive	lipoprotein, LysM	APL_1929	APP7_2018
55	APJL_1977		YP_001652969.1	soluble	weak positive	hypothetical protein	ND	APP7_2019
56	APJL_2004		YP_001652993.1	soluble	negative	hypothetical protein	APL_1957	APP7_2045
57	APJL_2060	*hbpA2*	YP_001653049.1	soluble	positive	heme-binding protein A	APL_2010	APP7_2097
58	APJL_2094		YP_001653083.1	soluble	positive	hypothetical protein	APL_2043	APP7_2130

[a.] The information of lipoproteins in JL03 is listed here, except two already studied lipoproteins Lip40 and PalA[1,2].

[b.] The immuno-reactivity of lipoproteins were evaluated by western blotting, using anti-A. pleuropneumoniae serovar 7 antibodies as primary antibody.

[c.] A total of 47 proteins were expressed successfully in Escherichia coli, including 37 soluble and 10 insoluble proteins.

[d.] NT, not tested.

[e] ND, homolog was found in the target genome.

1. Hu, X. *et al*. Identification and characterization of a novel stress-responsive outer membrane protein Lip40 from *Actinobacillus pleuropneumoniae*. *BMC Biotechnol* **15**, 106 (2015).
2. Liu, C. *et al*. Be aware of immunogenic but not protective antigens: the *Actinobacillus pleuropneumoniae* PalA as an example. *Protein Pept Lett* **24**, 1059–1065 (2017).
